# Ritualized Law and Livelihood Fragility of Left-Behind Women in Rural China

**DOI:** 10.3390/ijerph17124323

**Published:** 2020-06-17

**Authors:** Chao Wang, Jiayi Tang

**Affiliations:** School of Public Policy & Management, China University of Mining and Technology, Xuzhou 221116, China; wangchaoccnu@163.com

**Keywords:** left-behind women, livelihood fragility, ritualization, family separation

## Abstract

Family separation in rural China has led to a considerably large number of left-behind women who have to deal with livelihood fragility. The Department for International Development (DFID) framework focusing on households provides a base to understand the livelihood fragility of these women. Based on this framework and the existing field research, this study identifies that the national macro-strategy of unsynchronized development of industrialization, informatization, urbanization, and agricultural modernization leads to a separated migration model for rural families. Furthermore, the process of social modernization increases the fragility risk of how the left-behind family functions. The traditional gender culture expectations also directly affect rural families to make the livelihood strategy choice of, “male working outside, female taking care of home”. Based on the above theoretical research, this study extracts the concept of “ritualized law” to shed light on gender differentiation and family separation. A number of formal social security institutions have been established to promote the development of farmers, however, the ingrained culture gender differentiation encourages men to work in the profitable urban industry while women work in the field of unpaid agriculture and shoulder the responsibility of housework. This makes the formal institution a symbolic ornament for left-behind women, while they are forced to stay in rural areas and suffer from the fragility of livelihood.

## 1. Introduction

**Figure 1:**
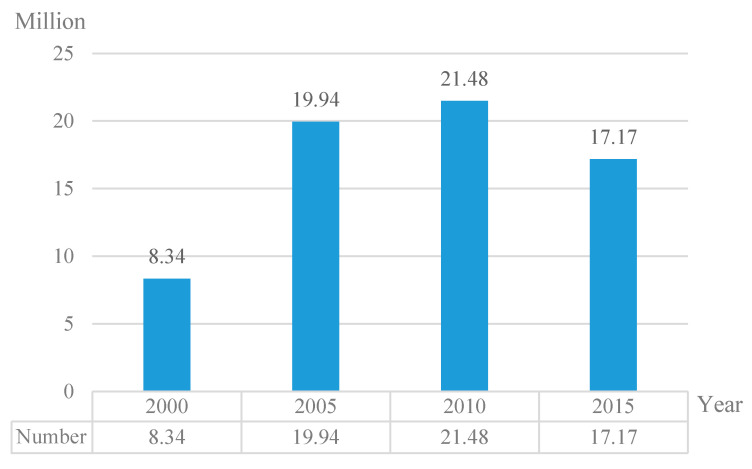
Annual distribution of left-behind women in rural China. Data source: Duan, C., Qin, M., and Lv, L. Study on the distribution and human development of left-behind wives in rural areas: An analysis of 1 percent population sampling survey 2015. *South China Population*. **2017**, 32(02), 34–49. Note: The left-behind woman here refers to adult woman aged between 20 and 59 who stay in the hometown while the husband works in a different city.

Industrialization, urbanization, and the relaxation of migration bans between urban and rural areas have collectively triggered the large-scale flow of migrant workers in China [1]. In 1995, there were 50 million migrant workers. Following that, the number of migrant workers has been growing rapidly at a pace of 6–8 million each year. China had more than 100 million migrant workers in 2003. There has been a steady growth in the number of migrant workers since 2003 and in 2011 it exceeded 250 million. In 2019, the total number was over 290 million. This movement of rural labor manifests a growing situation of gender differentiation and family separation. The gender composition of 2019 also demonstrates this fact. While the male labor accounted for 64.9%, the percentage of female labor was only 35.1. The movement of female rural labor is obviously less than that of male rural labor. Additionally, 80.2% of the migrant workers are married when they leave their homes. Cai (2018) reckons that almost 80% of rural families are in a state of family separation (Cai Fang (2018) calculated the proportion of “migrant workers going out with family members” and its annual average growth rate based on the national monitoring and survey report of migrant workers from 2010 to 2014, and the results indicate that only 13.3% of migrant workers move with family members) [2]. The increasing gender differentiation and family separation has led to a considerable number of left-behind rural women in China (Figure 1).

The term “family separation” in this paper does not imply family disintegration. It implies that the common life of nuclear family members in the same space is transformed into a separate common life in different spaces, because of which they cannot enjoy normal family life [3,4]. This indicates that after the husband leaves for work, the left-behind women have to shoulder the responsibility of family livelihood, face the difficulties of livelihood fragility alone, and their quality of life may fall below the normal level. The fragility of left-behind women’s livelihoods and the separation of their families are caused not only by the statutes of the formal system, but also by that of the informal system. Therefore, the analysis of the problem should be a dynamic and non-linear complex process, and the single analysis method and strategy would not sufficiently explain the background of the problem [5]. Academia has mainly studied these problems from two perspectives, the macro structural-institutional perspective and the micro family-strategic perspective [6]. These two perspectives provide a comparatively reasonable explanation of the problem, but the result may be biased if it is analyzed from one aspect alone.

It is important to constantly meet the growing needs of rural left-behind women to ensure that they have a better life and to make the sense of acquisition, happiness, and security of this group more substantial, secure, and sustainable. However, it is difficult to achieve sustained positive results through temporary rehabilitation strategies. There is a dire need to identify a fundamental solution to eliminate the fragility of rural left-behind women’s livelihoods based on methods that would also reduce the number of left-behind women. Based on survey data, this study adopts qualitative and quantitative research methods, following the Department for International Development (DFID) framework, to analyze the problem of livelihood fragility of left-behind women in rural China, and then attempts to respond to the following three main questions: Firstly, it gives a complete description of the cause of the fragility of rural left-behind women’s livelihood, which strongly proves that the crux of the problem lies in the separation of rural left-behind families. Secondly, it propagates that a logical analytical framework should be selected that links the macro and micro to explore the fragility of rural left-behind women’s livelihoods. Thirdly, it proposes that a logical integrated development framework should be created to end the problem of rural left-behind women, and effectively resolve the livelihood fragility of these women.

## 2. Conceptual Framework

To understand the concept of “livelihood fragility”, it is necessary to clarify the connotation of the terms “livelihood” and “fragility”. As a new paradigm in poverty studies, livelihood has received widespread attention from academia. Extensive research has been conducted within this paradigm, mainly with a focus on rural poverty alleviation and rural development [7,8,9]. Most scholars define livelihood as, “the way people make a living, which is built upon one’s capabilities, assets, and activities” [10]. The term “livelihood” has a richer context and wider extension than “work”, “income”, or “survival” does, hence, it can depict the living conditions of special groups in more detail. “Fragility” is a widely used concept, such as in phrases like “the fragility of body”, “emotion fragility”, “the fragility of price system”, and so on. Therefore, the understanding of this concept is a matter of opinion and conjecture. Among these understandings, the Department for International Development (of the UK) explains “fragility” from the perspective of risks that a person or his or her family encounter and the extent of damage that these risks bring about, as well as from the perspective of the ability that a person or his or her family have to alleviate or reduce threat, disaster, and various risks. The World Food Programme (WFP) believes that fragility is influenced by risk possibility, risk resisting ability, and the social service system. It is imperative to clarify that fragility does not refer to the state of shortage or deficiency, but the inadequate ability, insecurity, and the extent of damage when one tries to resist the risks, shock, and pressure that they face. Fragility is analyzed in this study to identify risks, strengthen livelihood, and consequently, avoid damage. The term “poverty”, which implies shortage or deficiency, is analyzed in the study to suggest measures to reduce poverty. From the above, livelihood fragility can be defined as the possibility of a person or his or her family to encounter risks and the extent of damage that these risks can bring about, or the possibility that a person’s living standards drop to a level that is below the acceptable level.

Chinese scholars estimate the fragility of livelihood on the basis of livelihood assets. This is mainly because the condition of the assets is usually the basis for a person’s choices in terms of opportunities, living strategy, as well as their risk environment [11]. Chambers and Conway (1998) segregate livelihood assets into two types: physical assets, mainly in the form of storage materials or resources, and intangible assets, which primarily refers to claiming rights and rights to access [10]. Moser (1998) believes that the ability to achieve different livelihood strategies is dependent on a person’s personal and family possessions of social and physical capital [12]. He further classifies livelihood assets into four categories, that is, “human capital”, “social capital”, “natural capital”, and “financial capital”. The DFID framework, developed by the Department for International Development (DFID), UK, groups livelihood assets into five categories, which are as follows: natural capital, financial capital, physical capital, human capital, and social capital [13]. 

**Figure 2:**
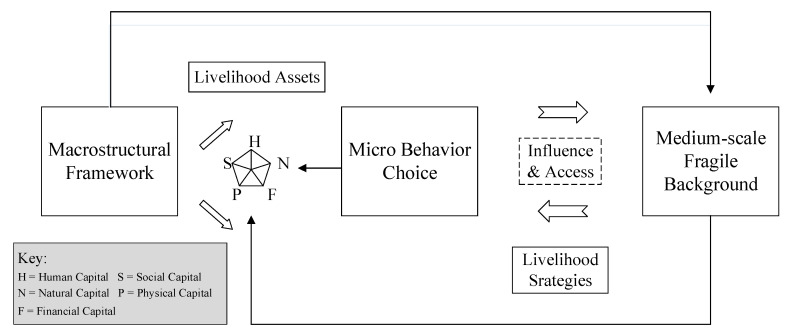
Analytical framework of the formation mechanism of livelihood fragility of rural left-behind women (made by authors)**.**

The DFID framework in Britain is the most widely used framework for the analysis of livelihood fragility [14]. This framework emphasizes on the different aspects of fragility that have been proposed by various international groups and institutions [15,16,17]. It outlines how vulnerable individuals and families pursue different livelihood strategies through livelihood capital portfolio in the context of structure and process, thus producing different outcomes and effect on livelihood capital, which facilitates in-depth study and reveals the generational logic of rural left-behind women’s livelihood fragility. It emphasizes both the ownership of the subsistence capital and the initiative of the choice of livelihood strategies, as well as the macroeconomic policy factors of the external environment. It also includes some less-related concerns, such as social shocks in the research context, laying the foundation for a comprehensive analysis of the problem. Presently, Chinese scholars mainly use the DFID theoretical framework to study the fragility of farmers’ livelihood [18] and to study the issue of rural left-behind women [19,20], which provide the appropriate basis and explanation for this study. Further, the DFID framework takes family as the research object, which coincides with our research that considers “family separation” as the starting point of the analysis (in fact, existing studies have suggested the need to improve the DFID framework by introducing the perspective of micro-demographics and integrating it with other livelihood elements to better study the decision-making mechanism of livelihoods in rural China. [21]). Therefore, this study uses the DFID framework to analyze the issue of livelihood fragility of left-behind women in rural China from the perspective of family separation (Figure 2).

## 3. Research Methods

This research adopts a qualitative and quantitative study to analyze the problem of rural left-behind women’s livelihood fragility and its formation logic. A questionnaire survey and semi-structured interviews were used to collect data. The questionnaire consists of nine parts, including family economy, physical health, children education, agricultural production and occupation, family and social networks, emotional communication, mental health, entertainment and self-development, rights protection, and women’s congress construction. The questionnaire also includes the necessary social demographic variables, namely age, education, number of children, occupation, years of marriage, number of long-term residents in the household, and annual household income. The questionnaires were given to nine hundred people and 812 valid questionnaires were collected, thus, the collection ratio was 90.2%. The questionnaire investigation method has been used to create an overall image of left-behind women’s livelihood fragility, as well as to determine the crux of the problem. The semi-structured interview has been used to attempt to vividly depict the living conditions of these women with baseline data and to prepare empirical information for investigating the problem of family separation. Based on the questionnaire, we visited 53 villagers and obtained substantial baseline data for analyzing the status quo and the causes of their livelihood fragility. 

Hubei Province was the place of the survey, and the following facts were taken into consideration. As one of the major provinces in central China, Hubei connects not only the north and the south but also the east and the west. The flow pattern of rural laborers here is a combination of trans-provincial flow and inter-provincial flow. The large migrant population of workers and the large number of the left-behind population here offered substantial resources for our survey. In 2011, there were 6.2938 million rural male migrant labors in Hubei, of which 3.5245 million were married. These workers left-behind 2.368 million women, which accounted for 67.2% in all married migrant families. This implies that most migrant families live in a state of marital separation and 2.368 million families are “separated common family living bodies”. To confirm the macro data, six villages from six cities were randomly selected to conduct a survey. It was observed that migrant families made up more than 65% of the total rural households and the percentage of families with both husband and wife working in cities is about 25, while families with just the husband working outside accounted for about 60% in the migrant families (Table 1). This indicates that most migrant households in the village are in a state of marital or semi-marital separation. Upon comparing the above macro and micro data, it is evident that the two reflect the same status. This further confirms that there are more than 60% of migrant rural families now facing the problem of marital separation. 

While selecting the survey location, the following factors were taken into consideration: the differences of urban class, size, urbanization level, geographical position, and the number of left-behind women [22]. Six cities were identified to conduct the survey. Geographically, the survey covers the east, center, north, and west of Hubei province. In the first stage, a large-scale questionnaire survey has been adopted, and stratified multistage sampling, random sampling, and normal control and baseline interviews have been used to thoroughly investigate the living conditions of rural left-behind women. The same method has been used to select appropriate respondents. Based on the first stage, the second stage applied a filed survey and depth interview focusing on the key research object (Table 2). This provided a profound insight regarding the hardships faced by the left-behind women and the crux of this problem. 

Well-trained investigators majoring in public administration, from China University of Mining and Technology, were responsible for collecting research data through face-to-face interviews using structured research questionnaires and for conducting in-depth interviews with respondents. In the formal survey, all respondents were given a brief introduction on this research and instructions for filling out the questionnaires, then respondents were required to independently and anonymously complete the questionnaires. Each questionnaire took about 20–30 min to fill out, each depth interview took about 30–40 min, and all respondents were offered compensation for their time-consumption. Further, trained investigators were assigned to read out questions in the questionnaires for those respondents who had difficulty reading, and check the questionnaires for implausible responses or missing data before the analysis of questionnaires. 

All the information collected through face-to-face interviews and questionnaires was confidential and anonymous. Written informed consent was obtained from all participants and it clarified that participating in this research was fully voluntary. In addition, since most of the interviewees had difficulty expressing, and their Mandarin was also not very standard. During the interview, their expressions were often mixed with dialects and unclear sentences. We optimized the expressions of interviewees without changing their original intention for better readability and clarity.

## 4. Main Findings

### 4.1. The Livelihood Fragility Problem Faced by Left-Behind Women 

Among the elements of the concept of livelihood, livelihood capital occupies the core position. Therefore, livelihood capital is basically used as an evaluation index in academia. In this research, the separation phenomenon of rural left-behind families leads to some constraints in family structure and function, which makes left-behind women face the plight of livelihood fragility, which is manifested in the following five types of livelihood fragility.

The ideological germination of social capital can be traced back to Durkheim’s “collective consciousness” and Zimmer’s “reciprocal exchange”. However, as a theory, it began with the study of Bourdieu, Parson, and others. After Coleman’s theoretical definition of social capital, Putnam, Porter, and some others, further developed the theory of social capital. In the DFID framework, social capital represents the social resources that people can use in the pursuit of livelihood goals. After the husband moves away for work, the left-behind wives in the countryside have to adapt from being the “housewife” to functioning as the “backbone” of the family. They have to shoulder all the family responsibilities that were originally shared by the husband as well, and also have to deal with the resulting physiological and psychological pressure. Although these women often feel powerless, they are afraid to seek help from other males. The rural society is an acquaintance society. The closeness and traditions of the society are very strict regarding the role of women and their morality. Consequently, left-behind women have to avoid any gossip that could connect them with other men. According to the survey, 26.4% of the left-behind women were the subject of gossip while their husbands were away (among these left-behind women, 75.4% were between 31 and 50 years old, 94% gave birth to one or two children, 61.4% were separated from their husbands for more than six months, and 64.8% of these families had the per capita annual income of less than 5000 RMB) (Appendix A). Mrs. Wang’s experience illustrates the embarrassing social interaction dilemma of these women:

Mrs. Wang, whose husband went to work in the city after she gave birth to their second child, had to take on the farm work, while also taking care of her parents-in-law and her two children. During the agricultural season, she can only ask relatives or neighbors for help, because she is afraid that people would gossip about her if she asked other men for help

*(personal communication).*

The production process of social capital is a process of communication and connection between people, people and organizations, and organizations and organizations. The scope of social contact of left-behind women is limited, and is only based on blood and geographic relationship that is maintained between relatives and neighbors. The concept of “virilocal marriage” also weakens, or even breaks down, the existing social network of left-behind women, and their social scope presents an “involution” situation. Additionally, 92.5% of the left-behind women have to engage in agricultural production, and also spend an average of 5.71 h a day in family care and support. This undoubtedly further restricts their participation in public life and affects their social activities. This participation is not only a wake-up call to expand political participation, but also an important way and means to accumulate social capital. Left-behind women have no time to cross-examine politics and their capacity to contribute toward social capital is also reduced because of over-involvement with the family and poor social capital. However, there is no denying that left-behind women are the link between urban and rural areas. The husband shares the stories in the city with his wife, and then the left-behind woman shares them with relatives in the rural home, which promotes the communication between the husband and the rural family.

In the 1950s, American economists Sehultz and Becker regarded personal education and vocational training as an investment, which would form personal “human capital”. In the DFID framework, human capital represents knowledge, skills, abilities, and health status. These are means for people to pursue and achieve different livelihood goals. The questionnaire survey shows that 96.1% of the left-behind women have children, which implies that taking care of them and educating them becomes their responsibility. However, only 16.8% of these women have high school diplomas or higher education. Consequently, these women cannot support the education of their children adequately, hence, 61.9% of these women are concerned about the learning of their children. Furthermore, only 5.8% of these women often participate in training on children’s education. The lack of education and the cultural background also affects their ability to grasp information related to agricultural science and technology. Consequently, they cannot be a part of the agricultural technology team and can only engage in traditional manual labor which offers low added value. Mrs. Zhao’s attitude reflects the lack of understanding science and technology by left-behind women:

Mrs. Zhao, a left-behind woman with only junior high school education, ploughs seven acres of land by herself. She thought that this work was very hard and did not understand the new technologies that could be used for ploughing. Also, she did not want to try using new technologies or new methods, as she was afraid that she would not be able to deal with the loss if she made a mistake

*(personal communication).*

Playing the role of “both housewife and backbone” not only made it difficult for these women to have the energy, time, and motivation to participate in agricultural technology training, but also immensely increased the pressure on them and, hence, led to physiological and psychological problems. The surveys and interviews reveal that continuous physical labor, especially when there were children to take care of, was having an adverse impact on the health of these women. Sixty-four percent of these women stated that their physical health is not ideal. However, most of these women ignore their health and do not get treatment as is needed. This is evident from the fact that 44.6% of these women do not meet a doctor when needed. Only when they did not have a choice, 54.6% of them chose to see a doctor. The family economic conditions of these left-behind women were generally poor, the survey data shows that more than 65% of these families had the per capita annual income of less than 5000 RMB. As the pillar of the left-behind family and the main force of rural daily production and life, their health problems do not only have an impact on just themselves but also on the entire family and even the rural society. The following case illustrates the health risks associated with their attitude:

Mrs. Xu is a left-behind woman, and she is always busy with housework and agricultural work, hence her health suffers. She often buys medicines from the village peddler, which functions as an informal pharmacy. She says, “The medicines are quite cheap, and it saves me the trouble of going outside the village. At least this medicine helps a bit, this is better than taking no medicine at all”

*(personal communication).*

In the framework of DFID, financial capital is the accumulation and flow that people require to fulfill their livelihood goals through the process of production and consumption. It refers to money, as well as to the physical objects that can play a role in the accumulation and exchange of money [11]. Jin et al. (2011) defined financial capital as available savings and regular capital inflows [23]. A lot of rural labor force has been shifting to the cities, since the 1980s and the trend of masculinity implies that a large number of women stay in the countryside, as the original, “male farming and women weaving” division of labor in the family has been gradually replaced by “male working and women farming”, and the phenomenon of the feminization of agriculture is becoming increasingly common. However, this cannot be precisely termed as the feminization of agriculture as women’s agriculturalization, because in reality it is a situation of “women working while men manage”, and the decision-making power regarding agricultural production and operations is still controlled by men. In this survey, 68.5% of the left-behind women could not make important decisions pertaining to the family, agricultural production, and life independently. Even though 61.4% of these left-behind women had been separated from their husbands for more than six months, they still could not make important decisions at home and in their agricultural activities (Appendix A).

Additionally, the relative weakness of the agricultural economy makes the family contribution rate of the left-behind women also decline. According to the survey data, 80% of the left-behind women accounted for less than 40% of the total household income, and 44.2% of the left-behind women accounted for less than 20% of the total household income (Appendix A). Even if about 30% of these left-behind families had the per capita annual income of more than 5000 RMB, the family economic contribution of the left-behind women was still quite limited. Additionally, as the husbands do not live with the family and the women have limited time and energy, the lack of a proper educational environment is expected to have a negative impact on the behavior and values of left-behind children. Taking this into consideration, most left-behind women consciously spend more time and energy on their children. Survey data indicates that the average time spent on housework by these women is 2.47 h per day, the time for educating their children is 1.68 h per day, and for caring for the elderly is 1.56 h per day. However, as women are not remunerated for the housework, their contribution is neglected. 

In the DFID framework, material capital includes infrastructure that is required to support basic livelihoods. Jin et al. (2011) regard communication and information services that farmers can afford, as material capital [23]. As the level of education of left-behind women is low, it is difficult for them to update their internalized agricultural knowledge and master new agricultural technology, which affects their income-generating ability. This ability is also restricted as the women do not possess the skills to collect and analyze information. According to the survey, there are limited channels for left-behind women to obtain information and consequently, 45.3% of these women urgently need market information services. This could also be attributed to the fact that these women have limited free time and energy to collect and analyze information. Additionally, they do not know how to use media to obtain information, and it is difficult for them to grasp the market information in time. Consequently, they cannot meet the production requirements effectively according to the market demand, and miss out on the opportunity to make a profit (in reality, agricultural products have another destinations except for the big markets, that are, self-sufficiency or in local markets, and that even account for a large proportion. However, women have no, or just a little remuneration, for this part, so their contribution is always neglected).

Due to the formation of the agricultural production labor market and the help of relatives, the agricultural production responsibilities undertaken by left-behind women have been reduced, but they still feel powerless when faced with temporary and sudden heavy manual labor. Additionally, Ren et al. (2011) believe that material capital primarily includes housing and family-owned facilities and equipment [24]. Most rural houses are relatively independent and far away from each other, and many of them have low courtyard walls and small gates. Criminals can easily enter the courtyard, which gives them easy access to the houses. Additionally, the outflow of the young male labor force from these areas has weakened the public security system. The physical and property security of left-behind women is vulnerable to infringement and this increases their psychological burden. These women are afraid of being alone at night because of the unsafe living environment in the countryside. Some of them even sleep with the TV set on as they are afraid of sleeping without any light or sound. The lack of material capital led to Mrs. Chen’s misfortune:

Mrs. Chen lived alone with her three-year-old daughter and one-year-old son. On the night of the incident, Mr. Li intended to only steal, but when he realized that Mrs. Chen and the two children were alone at home, he raped Mrs. Chen. Mrs. Chen did not inform her husband or the police because she was worried that her husband would file for a divorce and she was also afraid that she would be discriminated against by others

*(personal communication).*

Natural capital refers to the storage of natural resources and environmental services from which livelihood-friendly resources can flow and services can be derived. Presently, China’s per capita arable land area is 1.5 acres, however, the population has increased by 1.4 times, which implies that, in the absence of significant improvement in production conditions, there will inevitably be a conflict because of more people and less land. When a large portion of the male labor force in rural areas gives up working in the agricultural field, it eases the pressure on the family to a certain extent, and makes the left-behind women the backbone of agricultural production. However, because of the disintegration of the people’s commune, the focus on the construction of farmland and water conservation has decreased. This also makes it difficult to concentrate human, material, and financial resources on large-scale construction of farmland and water conservation facilities, and then the original farmland and water conservation facilities are not maintained or even suffered human destruction. In order to maintain normal agricultural production, much manual labor must be invested to meet the needs of land irrigation, however, due to the inherent physical disadvantages of left-behind women, they may not be able to afford the heavy manual labor, which makes it difficult for agricultural production to resist natural disasters due to the weakening of the protection. Consequently, agricultural families may fall into poverty (although economic remittances from husbands who have gone to work in urban cities have a significant contribution to improving the family’s economic situation, only a part of the rural families can get rid of poverty. For more rural families, on one hand, due to the high cost of living in the city, not all income of husbands can be sent home. On the other hand, children’s education and medical treatments require a great deal of money, which often leads to family expenditures greater than family income, and ultimately the families still have difficulty getting out of poverty).

The society in the rural areas in China is still largely agriculture centered. Land resources and property rights system regulate the process of the traditional society moving toward becoming a modern society. As Du (2005) said, “Land reform lays the foundation of today’s rural areas” [25]. With increasing modernization, comprehensive rural reforms in China are faced by a dilemma, and conflicts of interest have intensified. Amongst these, gender discrimination in rural land allocation has gradually changed from being recessive to explicit. This is visible on two axes [26], which are centered on gender identity, targeted at rural women and characterized by the deprivation of gender land rights and interests. This is manifested as rural women’s right to land contractual management, the right to use residential land, the income of collective economic organizations, and the right to allocate land expropriation compensation fees, being infringed [27]. Left-behind women who are categorized as exogamy women are facing the dilemma of “two ends empty” that “their mother’s land is recovered and their mother-in-law’s land is not available to them”. Some of them cannot claim the right to contract in their mother’s family, and some lose the right to contract and manage because of divorce and widow remarriage. Mrs. Zheng is one of them who had to deal with this awkward situation after she got married:

When Mrs. Zheng, a left-behind woman, submitted an application for land for personal housing due to demolition and reconstruction, she was refused and she was informed that she could not be included in her parent’s residence registration anymore because she was married. At the same time, her husband had not registered her name either

*(personal communication).*

### 4.2. Macro-Structure Framework: Modernization Construction with Unsynchronized Development of Industrialization, Informatization, Urbanization, and Agricultural Modernization

As an external policy environment, livelihood system includes the entire process of livelihood capital production, livelihood strategy selection, and livelihood outcome output. The scattering of rural left-behind families is the response of farmers to the asynchronous modernization of industrialization, informatization, urbanization, and agricultural modernization, and the result of the choice of family livelihood strategies of the husband going out while the wife stays back. The essence is that some vulnerable groups are forced to break away from the mainstream of development. This is the negative spillover effect of the state’s attempt to rapidly build industrial modernization through the effective use of limited public resources.

**Figure 3:**
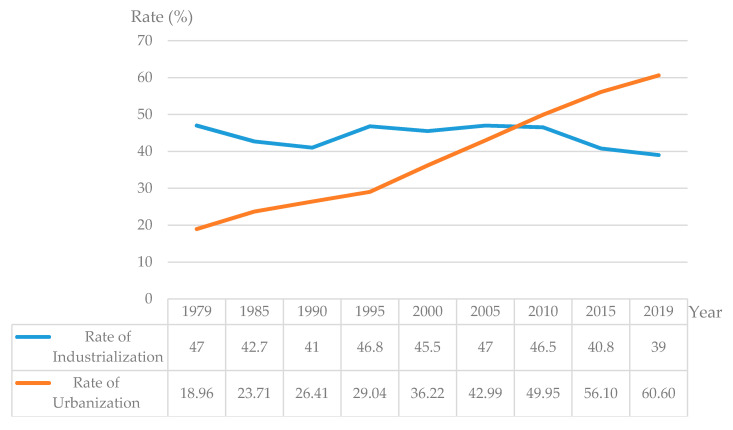
Rate of industrialization and rate of urbanization from 1979 to 2019 in China (Data source: National Bureau of Statistics).

First, the relatively slow urbanization compared to the pace of industrialization inhibits cities from providing a conducive environment for the migration of the entire family rather than just the migrant workers. China’s modernization with industrialization began in 1949. To ensure the smooth progress of modernization with industrialization as the core, the state established certain urban preferential policies with the household registration system as the core content. To begin, these are intended to build a good social order, and then gradually evolve into an important means of social control, resource allocation, and benefit redistribution, to prevent the large-scale flow of rural labor force. This leads to the stagnation of the movement of rural labor force and the process of urbanization. The rate of urbanization has gradually increased from 10.64% in 1949 to 17.92% in 1978. After 1949, as the country vigorously promoted the household registration system and relaxed the requirements of the household registration migration policy, large-scale rural labor began to relocate to cities and towns, and the urbanization rate increased significantly. In spite of this, a very large gap still exists when compared with the long-term industrialization rate of about 45% (Figure 3). The rate of urbanization has been lagging behind industrialization for a long time, and this makes it difficult for cities to provide infrastructure and basic public services for migrant workers, and forces them to move between urban and rural areas in the form of family separation

Second, the disharmony between urbanization and agricultural modernization lead to low agricultural comparative benefits and imbalanced urban-rural public services. Although “industry feeds back agriculture, city feeds back countryside” is now a consensus, it has not fundamentally changed the interest pattern of unbalanced industrial and agricultural development and has distorted urban-rural market relations in China. This symbiotic state of preference has two impacts. First, agricultural economic income is relatively low as the economic added value of agriculture is far less than that to the industry and service industry, it is difficult for farmers to earn a higher income solely from traditional agricultural production. The income gap between urban and rural areas in China has gradually widened since 1978. By 2017, the ratio of disposable income between urban and rural residents had reached 2.71. To address the economic interests, the movement of rural labor force to cities is inevitable. Second, the basic public services in rural areas are not well established. The long-term implementation of the “agriculture supporting industry, rural supporting urban”, development method has led to a lag in the development of rural infrastructure and basic public services. Due to the lack of a proper supply system for rural family production and life care services, migrant workers can only rely on their own human capital to address the lack. Therefore, based on the strategy of “working in cities and farming in countryside” and the necessity of family care, wives continue living in the countryside to take care of the families and agricultural production, while husbands have to leave the home to work in cities and towns.

**Figure 4:**
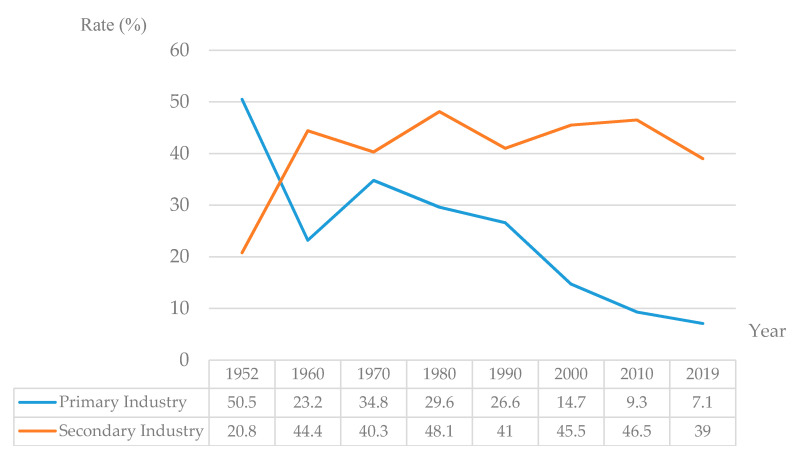
The rate of primary and secondary industry in GDP (Data source: National Bureau of Statistics).

Third, the lag in agricultural modernization as compared to industrialization makes it difficult for rural laborers to work in their hometown. After the founding of the People’s Republic of China, the national strategy of priority development of heavy industry was implemented. Industrial development funds were accumulated through the “scissors gap” price of industrial and agricultural products, and raw material needed for industrial development were continuously drawn from agriculture. After 1979, the rural surplus labor force, got the opportunity to move to the city. As industries drew a large number of cheap laborers from agriculture they made great progress. From that time on, the positioning of agriculture and industry in the national economy has undergone tremendous changes. In 1952, the primary industry accounted for 50.5% of the GDP, which implied that it was a dominant part of the national economy. By 2019, the proportion had dropped to 7.1 percent. Conversely, the proportion of secondary industry in GDP in 1952 was 20.8%, and it rose to 39% in 2019 (Figure 4). Agricultural modernization and industrialization have existed in this parasitic state for a long time, which implies that industrialization is not strong enough to drive agricultural modernization and rural industrialization, and the ability of rural surplus labor work force to transfer directly to secondary and tertiary industries is limited. Driven by comparative interests and rational choice of urban and rural employment, a large number of rural labor work force flowed into cities, resulting in the abnormal structure of the “separation” of rural families.

Fourth, the ubiquitous and parasitic nature of information technology enables its integration with industrialization, agricultural modernization, and urbanization, and it acts as a symbiotic interface for material, information, and energy exchange between the other “three modernizations” and the external environment [28]. However, due to the insufficient development of informationization and its insufficient integration with the “three modernizations”, it is difficult to promote them to the advanced stage of development based on information resources, driven by knowledge innovation and carried by the Internet, so that urbanization and industrialization have insufficient driving effect on agricultural modernization. Agricultural informationization can reduce agricultural water consumption by 30–70% and improve land use efficiency by more than 10% by adopting precise water-saving irrigation technology [29]. Additionally, the soil moisture in the topsoil can be improved, and winter wheat yield can be promoted by adopting laser-controlled land leveling technology [30]. However, the integration of informationization and agricultural modernization in China is insufficient, and the level of informationization is relatively low. It is still at the early stage of growth [31]. As stated previously, the asynchronization of industrialization, urbanization, and agricultural modernization has led to the separation of rural left-behind families. However, the lack of integration of informatization and the other three modernizations aggravates the asynchronization of the development of industrialization, informatization, urbanization, and agricultural modernization, which indirectly has in impact on the separation of rural left-behind families.

### 4.3. Medium-Scale Fragile Background: The Social Modernization Process of Continuously Weakening Families

In the DFID framework, fragility background refers to the external livelihood environment that people have to deal with, which fundamentally affects their livelihood and the available livelihood capital, especially the economic benefits of their livelihood activities and the corresponding choices. In the process of social modernization, the values of family reunions are undermined, there is a negative impact on the traditional organization of family relationships, and it disintegrates the normal functions of family. Consequently the traditional gender differences in rural areas and the urban-rural dual household registration system jointly resulting in the families of farmers rationally making a choice regarding the livelihood strategies based on gender differentiation and migration based on family decentralization. Women are forced to stay in the countryside and have to deal with the resulting livelihood fragility alone.

**Figure 5:**
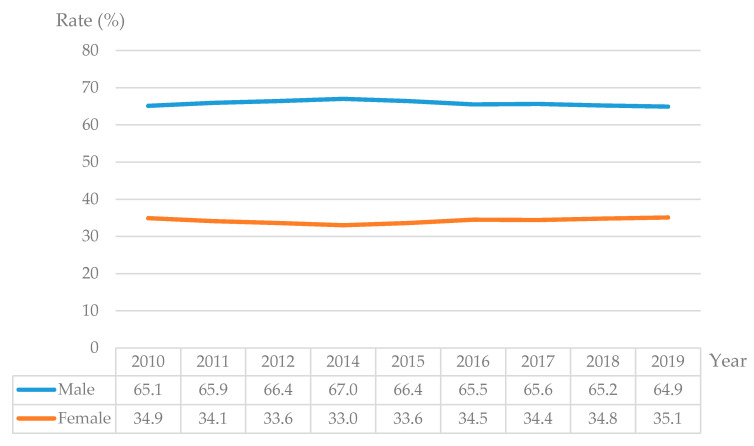
Rate of male and female migrant workers from 2010 to 2019 in China (Data source: National Bureau of Statistics. The data for 2013 is missing because the National Bureau of Statistics did not publish the rate of male and female migrant workers in China).

First, the family spirit is the Constitutional spirit of China, but the process of social modernization is a process that constantly impacts the traditional family consciousness [32]. From the Revolution of 1911, the New Cultural Movement to the May 4th Movement, the feudal family system became the critical object of the enlightenment thinkers, and the war and revolutionary idealism in the old and new democratic revolutions even antagonized the family and the country. In the last 30 years the movement from New China to reforms and opening up, social movements have constantly destroyed the traditional family model on an economic basis and ideological level. The extreme politicization of the “Cultural Revolution” lead to excessive harm to the family structure. Just as people dispelled the mistake of trampling on family values during the Cultural Revolution, the tide of reform and opening up swept over. With this, the basic unit of a modern society has been implemented to the greatest extent on independent people. The ideal example of this is the use of urban-rural dual household registration system to screen individualized, non-family-led rural male labor force, and the refusal to grant them citizenship to share urban welfare resources. Consequently, it becomes difficult for families of migrant workers’ families to establish themselves in cities and live and work with peace and contentment. Migrant workers can only migrate to cities in the form of gender differentiation, which is reflected in Figure 5:

Second, after the reform and opening up, the individual-based values spread in the society. It became difficult for families which were at the edge of the national vision to become the beneficiaries of system design and arrangement. The function and importance of the family has been increasingly ignored by the society, which lead to a further decline in the value of family in the design and arrangement of the national system. This implies that the basic rights and interests of migrant workers’ families in cities are not guaranteed and their demands are left unsatisfied. In this survey, 70.1% of left-behind women think it is expensive for their husbands to rent a house in cities, and 47.3% think it is expensive for their husbands to see a doctor in cities. Further, only 37.7% of left-behind women said their husbands had work-related injury insurance, 29.1% said their husbands had unemployment insurance, and 18.7% said their husbands had a housing provident fund. This forces migrant workers to attempt to “maximize family interests”. These workers try to avoid risks. Consequently, they have to make rational choices based on gender differentiation and family decentralization. Women are forced to stay alone in the countryside and bear the livelihood fragility caused by family separation. A large number of rural left-behind families and women have become victims of urban construction, and are unable to enjoy the benefits of a complete family. 

Third, the current practice of unbalanced modernization of urbanization, industrialization, and agricultural modernization reflects the government’s focus on economy and efficiency. This is essentially a developmental thought process which propagates the thought, “economic growth overwhelms everything” [33]. This thought process equates modernization with economic development, technological progress, and wealth increase. Public policy also propagates the principle of efficiency first. It responds to the demands of peasants by means of policy tools of management rather than service [34]. It primarily lays emphasis on the development of a material economy and neglects the value care that people need, and neglects that the fundamental principle of ensuring the well-being of the citizens should be development. This pathological concept of excessive focus on the development of modernity will lead to the development of modernization by overstepping the limited rationality, and will lead to neglecting the needs of people, gender differences, family disintegration, and the rural female labor force would have to live in the countryside to make a living. Village head Li reflected the neglect of left-behind women’s interests in the process of economic development:

Some left-behind women went to work in nearby factories, but most factories did not sign legal labor contracts with left-behind women, nor did they purchase necessary insurance for left-behind women. When left-behind women were injured at work, they only got medical expenses from the factory but no legal insurance compensation

*(personal communication).*

### 4.4. Micro-Behavioral Choice: Choice of Family Livelihood Strategies under Social Gender Culture

With the rapid advancement of urbanization in the country and the dual economic and social structure which gradually leads to the integration of urban and rural areas, a series of social security systems, such as the basic pension insurance system and the basic medical insurance system, as an important part of China’s economic and social structural arrangements, are also moving towards integrated development to achieve inclusiveness and equalization of public services. However, in the context of economic growth and over all development, the government focuses on economic development performance and other work that can indicate political achievements, and pays less attention to social equity, especially gender issues, and largely believes that the development goal of gender equity is pursued by a postmodern society. Furthermore, even if gender equity is taken into consideration while formulating policies, most of the focus is on protecting the rights and interests of urban women, and less on rural left-behind women.

Additionally, the livelihood strategies include production activities, investment strategies, and reproduction choices. The livelihood strategies in the farmers’ daily life practice also begin with the psychological framework used to understand the situation. Consciousness cannot be considered without its context, and every situation contributes toward the meaning of accumulation to structure the next moment. To a large extent, life takes place in a world constructed by human beings, where life has historical accumulation, which is an attitude toward the world [35]. The traditional gender division of labor in China has stereotyped women as being family-oriented, this also gives men priority in resource allocation, and the opportunity to enter the profitable urban industries while women are left behind to handle the unpaid family agriculture.

First, the Chinese traditional gender culture originated from the prevailing Confucian culture of patriarchy which was centered on male superiority and female inferiority. Despite the constant impact of social progress, the pattern of “male working outside, female taking care of home” has become even stronger and can be seen in all sectors of the society. This culture also requires women to regard “traditional virtues” such as “carrying on the ancestral line” and “assisting husband and educating children” as important criteria related to life value [36]. It requires women to confine their activities and time to address and indulge in trivial family affairs. Traditional gender concepts also endow rural women with psychological characteristics such as inferiority dependence, weakness and timidity, and substitutional achievement motivation. They lack the courage to break through traditional family roles to work in the non-agricultural industries. They are willing to act as housewives and regard the achievements of their husband as the realization of their own values. Survey data shows that 41.1% of the left-behind women agree with the division of “male working outside, female taking care of home”, and 54% of the left-behind women believe that women will encounter more difficulties in working in the city, and then they voluntarily stay at home. Therefore, in the process of urban-rural migration of labor force, the traditional gender concept has an impact on the role image and family expectations. This usually results in the husbands going to the city to work while the wives stay at home.

Second, one of the basic characteristics of Chinese traditional agricultural life is labor intensive cultivation with an iron plough and cattle ploughing as the primary methods. Thus, through the division of labor within the family based on physiology, men take on the labor heavy and intensive field work, while women take on the less intensive housework, forming a division of labor mode of “male cultivating and female weaving” in which the physiological advantages of the two can be observed. After the creation of New China, rural women began to break away from the fate of “working at home in the kitchen” and actively devoted themselves to “socialist revolution and construction”, playing the role of “half the sky” in the entire rural production and life. With the socialization of production, social division of labor reduces the labor time and labor required for agricultural production, this leads to the engagement of left-behind women in heavy agricultural production, promotes the continuous transfer of surplus rural labor to non-agricultural industries, and also leads to a situation in which, “men going out to work, women staying in the countryside”.

**Figure 6:**
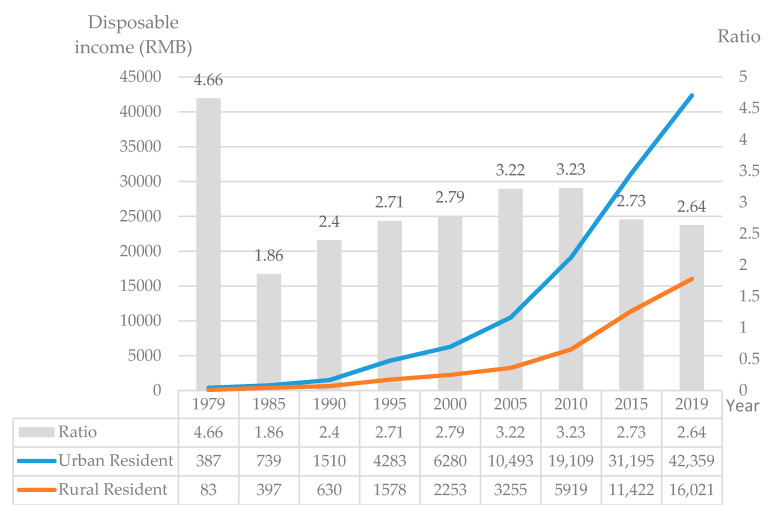
The annual disposable income of urban and rural residents from 1979 to 2019 in China (Data source: National Bureau of Statistics).

Third, the continuous increase in labor and agricultural prices offsets the growth of the farmers’ income, which largely reduce the enthusiasm of farmers to cultivate land, and also makes their income stagnant. Consequently, the income gap between urban and rural residents has not been greatly improved (Figure 6). To maximize family benefits, farmers prefer to work in cities rather than stay in rural areas. Furthermore, the dual household registration system in urban and rural areas requires that male elites, who meet the needs of individualization, have no family, and can bring the best benefits to the city should be screened out from the rural population. However, even if these eligible farmers move to the city, the urban social system still reflects their “peasant” status and excludes them from the welfare system. Therefore, in the attempt to “maximize family interests while avoiding risks as much as possible”, farmers tend to make a rational livelihood choice of “gender differentiation” and “family decentralization” migration.

## 5. Conclusions

In the process of modernization, it is a common phenomenon that labor forces from rural areas relocate to urban areas. This labors transfer is considered as the only road to modern economic growth. The “demographic dividend”, which implies the movement of the laborers from rural areas to cities, since 1978, is the largest movement of people that has ever happened in the world, and has created the prosperity of Chinese cities and is a key driving force for the country’s economic development. However, the modernization and development presently seen has led to unbalanced development of agricultural modernization as compared to urbanization, industrialization, and informatization. This reflects the government’s focus on economics and efficiency, which, in essence, implies that “Economic growth overrides everything” [33]. Accordingly, modernization is equated with economic growth, technology progress, and growth of wealth. Public policy, as a stable formal expression of this development practice, thereby accepted the rule of “efficiency comes first”, and adopted “managerialism” rather than “service-oriented” policy instruments to respond to public interest appeals made by rural residents. 

Under such circumstances, the local government is expected to advocate the pathological development logic, which is development for the sake of development, and invert the ends and means of modernization development. Development thereby inevitably exceeds the limit of development rationality. Human beings, gender differences, and family are all secondary in this kind of development. The typical manifestation of this is the adoption of dual urban and rural household register system to select appropriate rural labors for modernization construction in cities. This leads to the flow of male rural laborers to cities as they are independent individuals and do not have to take their families with them. At the same time, cities refuse to give these rural male laborers city membership with which they can enjoy the welfare offered as native city residents do. Consequently, rural residents make the rational choice of gender differentiation and family separation, that is, to achieve the goal of maximizing the entire family’s benefits while trying to avoid risk as much as possible, which leads to a large number of women being left behind in rural areas.

The issue of livelihood fragility of rural left-behind women can be explained not only through the starting point of “family separation”, but also through the “question window” to deeply explore the logic of problem formation, which can also reflect the status of China’s modernization. The theoretical framework of DFID focusing on family is to integrate the research on the above issues into a unitive logical framework, taking "family separation" as the starting point for analysis, and comprehensively and deeply reveal the livelihood fragility of the special groups and its generation mechanism. In this way, readers can understand the inherent logic of the theoretical framework of DFID and its interpretability in reality. Therefore, we follow the theoretical framework of DFID, in the integrated development framework of eliminating the phenomenon of family separation, to construct the governance method of the livelihood fragility of left-behind women by means of terminating “left-behind”.

From the perspective of the institution, although left-behind women in rural areas derive from family, it is attributed to the institutions. The family separation is essentially due to the failure of the institutions. From the dual urban and rural structure to a series of welfare institutions derived from it, like social security, education, and so on, a set of “perfect” formal institutions are seemingly built for farmers. However, the institution itself is a kind of resource, and who enjoys the benefits of the institution has to be determined. In practice, this often reflects a situation of gender imbalance. On one hand, based on the Chinese traditional gender concept, in which “men are superior to women”, the informal idea of, “male working outside, female taking care of home” has been solidified into a gender interest pattern that is now spreading and transmitting to all social aspects, and also shaping the traditional gender role of rural women. On the other hand, the socialization of labor production promotes the change of gender division of labor in the family, which enables the transformation of traditional “male farming and female weaving” into “male to work while female to farm” and makes it possible for left-behind women to engage in heavy agricultural production.

Against the background of the unsynchronized development of industrialization, informatization, urbanization, and agricultural modernization, the process of social modernization impacts the structure of the family and weakens the function of the family constantly, along with the influence of the rural traditional gender culture, the macro, medium, and micro factors jointly lead to the uselessness of the institutions for left-behind women, which generates the forced choice of rural families to maintain in the form of family separation and gender differences. Dovetailing with the wider theoretical and conceptual discussion on the nexus between formal and informal institutions, we extract the innovative concept of “ritualized law” to characterize the asymmetric relationship between formal institutions and informal rules. When informal rules are superior to formal institutions and affect their substantive functioning, especially when the institution execution mechanism is absent, formal institutions will be ritualized. The “ritualized law” makes the institutions only have the virtual symbolic meaning, but cannot play a role in policy practice.

The ingrained informal concepts and customs of male superiority and female inferiority takes it for granted that men would be going out to work, and the activities of women should only be limited to trivial housework. The traditional family’s gender division of labor has, in fact, transformed the formal and legal institution into a “symbolic ornament” [37,38], resulting in men going out to work in the urban industry to enjoy rights of priority in resource allocation while women work in unpaid family farming as housewives. This ultimately makes left-behind women a disadvantaged group that is ignored in the development trend and has to suffer livelihood fragility due to family separation.

Even if the legal and transparent formal institutions in the sense of Weber take precedence over other non-institutional rules, it cannot be denied that various informal customs and norms continue to coexist in the modern society, and these are crucial to shaping individual behaviors. These informal institutions create an environment where formal laws or institutions are merely looked upon as being full of reassuring words on paper and clauses, and serve a decorative role, as window-dressing lacking any real substance for left-behind women. Ritualized laws create an unequal world in which left-behind women have difficulty in pursuing benefits and legitimate interests, making them victims of the contest between the ineradicable developmental conscious inertia and the pursuit of fairness and justice. This can be equated with the scenario of survival of the fittest in which men with more resources and chances survive, leaving the left-behind women empty-handed. As long as the ritualized law still prevails, it will take a long time for China to develop from a country ruled by men to one ruled by law.

It should be taken into consideration that the ritualized law does not only exist in China. There are a large number of disadvantaged groups similar to the left-behind women in rural China all over the world due to similar laws, especially in developing regions. Most of these disadvantaged groups have difficulty in effectively achieving the acceptable quality of life, and if the situation does not change, it will be detrimental to the harmonious and stable development of the state. This implies that it is imperative to protect disadvantaged groups from livelihood fragility. To improve the lives of these disadvantaged groups, public authorities should disseminate ideas, norms, and cultural principals pertaining to gender equality and modern law through mass media, newspapers, and magazines. They must strengthen the equal consciousness of these people and the awareness of individual entitlements. The local government should also optimize the allocation of public resources and appropriately direct public resources to disadvantaged groups, and increase investment in public utilities and establish and improve the fully-covered social security institutions. It is necessary to improve the autonomous awareness of disadvantaged groups, and to establish autonomous organizations of these groups so that they can protect their interests with the support of these organizations.

## Figures and Tables

**Table 1:** Distribution of left-behind families in sampling villages.

Village	Total Number of Households	Total Number of Migrant Worker Households	Proportion (%)	Household in which Both Husband and Wife Work in Cities		Household in which Husband Works in Cities and Wife Stays at Home	
				Number	Proportion of the Number of Migrant Worker Households (%)	Number	Proportion of the Number of Migrant Worker Households (%)
Village Zhu	67	56	83.6	21	37.5	35	62.5
Village Xie	63	49	77.8	22	44.9	27	55.1
Village Liu	147	102	69.4	31	30.4	71	69.6
Village Huang	364	245	67.3	53	21.6	192	78.4
Village Tang	427	276	64.6	63	22.8	213	77.2
Village Mu	586	420	71.6	120	28.6	300	71.4

**Table 2:** Detailed information of interviewers.

Village	Number of Respondents	Sex		Age			
		Men	Women	20–30	31–40	41–50	51–60
Village Zhu	3	1	2	1	1	1	0
Village Xie	3	1	2	0	2	1	0
Village Liu	5	2	3	1	1	3	0
Village Huang	11	4	7	2	5	3	1
Village Tang	12	5	7	1	6	3	2
Village Mu	19	6	13	4	6	5	4
Total	53	19	34	9	21	16	7

## Appendix A

**Table A1:** Key variables of left-behind women’s livelihood fragility in China.

Variables	Percentage (%)	Age				Education					Number of Children				Separation Months with Husband		
		20–30	31–40	41–50	51–60	0	1–6	7–9	10–12	>12	0	1	2	>2	<6	6–12	>12
Gossip trouble																	
Yes	26.4	19.7	49.3	26.1	4.9	1.5	24.4	53.2	18.9	2.0	1.5	46.0	48.0	4.5	38.6	34.5	26.9
No	73.6	19.2	47.8	29.5	3.5	2.1	21.3	60.5	14.3	1.8	4.6	47.2	44.1	4.1	37.0	38.5	24.5
Health condition																	
Ideal	36	27.8	48.5	22.0	1.7	0.0	17.8	60.1	19.6	2.5	4.9	48.8	43.8	2.5	33.8	40.0	26.2
Medium	52.7	14.7	50.0	30.8	4.5	2.2	24.2	58.6	13.6	1.4	3.9	47.2	45.2	3.7	37.7	38.5	23.8
Bad	9.4	19.7	36.8	36.8	6.7	6.6	30.3	52.6	9.2	1.3	0.0	43.1	44.4	12.5	46.5	29.6	23.9
Extremely bad	1.9	25.0	25.0	43.8	6.2	0.0	31.3	68.7	0.0	0.0	0.0	62.5	31.3	6.2	73.3	20.0	6.7
Time for doing housework per day																	
1 h	16.5	33.6	39.6	26.1	0.7	1.5	19.5	56.4	21.1	1.5	6.1	57.3	34.4	2.2	39.2	40.0	20.8
2 h	38.9	18.4	49.0	27.4	5.2	1.6	20.9	59.5	16.3	1.7	4.0	43.7	46.7	5.6	48.1	33.9	18.0
3 h	25.6	19.8	51.2	26.6	2.4	3.0	24.1	58.1	11.8	3.0	3.5	46.0	47.0	3.5	32.0	40.5	27.5
≥4 h	19.0	11.1	47.7	35.3	5.9	1.3	26.8	59.5	11.8	0.6	1.4	49.3	45.8	3.5	23.4	40.0	36.6
Time for education children per day																	
1 h	48.7	16.0	48.6	30.4	5.0	1.9	24.4	56.2	15.4	2.1	3.5	46.2	45.7	4.6	40.1	36.2	23.7
2 h	37.9	25.8	50.7	21.5	2.0	1.4	21.2	59.0	16.7	1.7	4.9	45.8	45.1	4.2	35.7	41.5	22.8
3 h	10.5	16.9	45.8	34.9	2.4	2.4	18.1	66.3	13.2	0.0	0.0	61.5	38.5	0.0	21.8	39.7	38.5
≥4 h	2.9	39.1	30.4	26.1	4.4	4.3	13.0	73.9	4.3	4.5	4.5	59.1	27.3	9.1	60.9	17.4	21.7
Time for caring for the elderly per day																	
1 h	59.0	22.7	50.9	24.4	2.0	1.5	20.6	58.0	18.2	1.7	3.3	47.2	45.1	4.4	37.9	39.1	23.0
2 h	30.0	17.8	51.0	27.7	3.5	0.5	21.8	62.9	13.7	1.1	4.6	45.1	46.7	3.6	36.7	33.0	30.3
3 h	6.8	19.1	51.1	29.8	0.0	0.0	21.3	70.2	4.3	4.2	2.1	53.2	44.7	0.0	18.6	46.5	34.9
≥4 h	4.2	10.3	31.0	48.3	10.4	0.0	32.1	57.1	10.8	0.0	4.2	58.3	29.2	8.3	29.6	37.0	33.4
Proportion of left-behind women’s income in total household income																	
<20%	44.2	20.7	48.6	25.6	5.1	2.9	26.2	54.2	14.9	1.8	3.3	47.2	43.3	6.2	39.3	35.0	25.7
20–40%	35.8	15.5	51.8	29.9	2.8	0.7	21.1	59.3	17.9	1.0	5.1	44.0	48.4	2.5	33.3	42.2	24.5
41–60%	14.6	36.5	36.5	26.1	0.9	1.7	19.1	64.3	12.2	2.7	4.3	56.5	38.3	0.9	39.8	38.1	22.1
61–80%	3.9	6.5	45.2	45.2	3.1	3.2	19.4	74.2	3.2	0.0	0.0	50.0	43.3	6.7	32.1	46.4	21.5
>80%	1.5	8.3	58.3	33.4	0.0	0.0	16.7	75.0	8.3	0.0	0.0	50.0	50.0	0.0	54.5	27.3	18.2
Taking important decisions pertaining to the family, agricultural production, and life independently																	
Yes	31.5	19.8	46.2	30.0	4.0	2.0	20.9	62.1	14.2	0.8	4.1	46.7	45.9	3.3	35.1	41.8	23.1
No	68.5	19.9	48.6	27.6	3.9	1.7	23.6	57.1	15.4	2.2	3.8	47.8	43.8	4.6	38.6	36.1	25.3
